# Multispectral metal-based electro-optical metadevices with infrared reversible tunability and microwave scattering reduction

**DOI:** 10.1515/nanoph-2024-0202

**Published:** 2024-05-22

**Authors:** Zhen Meng, Dongqing Liu, Yongqiang Pang, Jiafu Wang, Tianwen Liu, Yan Jia, Haifeng Cheng

**Affiliations:** 58294 National University of Defense Technology, Changsha, China; Xi’an Jiaotong University, Xi’an, China; 66488 Air Force Engineering University, Xi’an, China

**Keywords:** metamaterials, electrochromic, infrared emissivity, multispectral, camouflage

## Abstract

The demand for advanced camouflage technology is increasing in modern military warfare. Multispectral compatibility and adaptive capabilities are increasingly desired features in camouflage materials. However, due to the strong wavelength dependence and limited tunability of electromagnetic wave responses, achieving simultaneous multispectral compatibility and adaptive capability in a single structure or device remains a challenge. By integrating coding metamaterials with infrared (IR) electrochromic devices, we demonstrate a highly integrated multispectral metal-based electro-optical metadevice. The fabricated metadevices enable the reversible tunability of IR emissivity (0.58 at 3–5 µm, 0.50 at 7.5–13 µm) and wideband microwave scattering reduction (>10 dB at 10–20 GHz). The excellent integration performance is attributed to the remarkable electromagnetic control capabilities of the coding metamaterials in a chessboard-like configuration and the IR electrochromic devices based on metal reversible electrodeposition. Furthermore, the monolithic integrated design with shared barium fluoride substrate and electrodes allows the metadevices to have a simple architecture, and the careful design avoids coupling between functions. Our approach is general enough for the design of various electrochromic devices and metamaterials for multispectral camouflage, offering valuable insights for the development of advanced adaptive multispectral camouflage systems.

## 1 Introduction

Organisms in natural ecosystems have evolved a variety of camouflage strategies to enhance their ability to blend in with their surrounding environment, hence facilitating successful hunting endeavors and minimizing the risk of predation. Similarly, in human society, strategies for detection and reconnaissance as well as for camouflage and stealth continue to advance one another. The integration of optoelectronic technology has led to the deployment of detectors across multiple wavelengths, posing a significant challenge for traditional camouflage methods. The demand for multispectral compatible camouflage materials has never been greater, particularly in the realm of infrared (IR) and microwave detection technologies, which are prevalent in modern warfare [1], [2]. However, there exists a fundamental contradiction between IR camouflage and microwave camouflage in terms of material absorption rates [3]. Consequently, the investigation of IR-microwave compatible camouflage materials has become an important research topic in the field of multispectral compatible camouflage, both domestically and internationally. Numerous proposals have been put forth in the field, such as combining metal powders with microwave absorbing materials [4], [5], covering microwave transparent patterned metal arrays with microwave absorbing structures [6], [7], [8], [9], and utilizing one-dimensional photonic crystal structures [10] for IR-microwave compatible camouflage. Nevertheless, current efforts are mainly focused on achieving static IR camouflage, aiming at low levels of IR emissivity. Hence, the efficacy of static camouflage measures is challenged when confronted with intricate and dynamic combat landscapes, as well as the need for weapons and equipment to navigate diverse geographical regions and temporal contexts. In recent years, there has been a growing focus on adaptive IR camouflage technologies within the field of camouflage stealth research [11], [12], [13], [14]. According to the Stefan–Boltzmann law, dynamic IR camouflage can be achieved by manipulating the temperature of the target surface or by modulating the emissivity of the surface. One potential way among the available options is electrically variable emissivity. The advantages of this technology include its flexible modulation capability, fast response time, lightweight construction, and low energy usage [15]. Among many electrically variable emissivity devices based on various mechanisms, reversible metal electrodeposition (RME) variable emissivity devices demonstrate notable advantages due to their large and uniform emissivity modulation capability achieved by manipulating the metal electrodeposition and dissolution processes [16], [17], [18]. Unfortunately, various electrically variable emissivity devices, including RME variable emissivity devices, are easily detectable by radar due to the high microwave reflectivity properties of the conductive electrodes in the device structure, thus limiting further compatibility with microwave camouflage.

**Figure 1:**
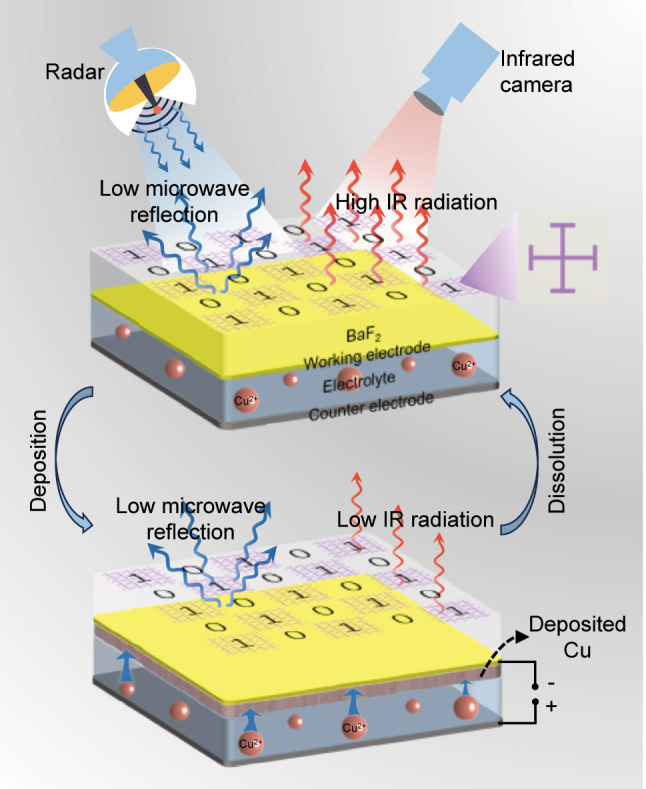
Schematic illustration of the proposed metadevice with electrically tunable IR emissivity and microwave scattering reduction. The metadevice exhibits high IR radiation in the dissolved (initial) state. When the deposition voltage (−2.5 V) is applied, the active metal ions Cu^2+^ in the electrolyte are reduced and deposited on the working electrode, forming a layer of Cu electrodeposited Pt film, switching the device to the deposited state with low IR radiation. When the dissolution voltage (2.5 V) is applied, the deposited Cu on the working electrode is oxidized, causing the metadevice to switch back to the dissolved state. Integrated with coding metamaterials with a chessboard-like configuration, the metadevice consistently exhibits low microwave reflection during the whole electrodeposition and dissolution process.

Electromagnetic metamaterials, as a new type of artificial electromagnetic materials, can realize exotic electromagnetic properties that are difficult to be achieved by natural materials through specific arrangement and combination of periodic sub-wavelength structural units [19], [20], [21], [22]. Coding metamaterials represent a burgeoning field within electromagnetic metamaterials that incorporates the concept of digital coding into metamaterial design [23], [24]. This involves coding structural units with distinct phase responses, enabling the flexible manipulation of electromagnetic waves through the design of coding sequences. Consequently, research in the field of microwave camouflage has been advancing based on coding metamaterials [25], [26]. In this study, we present a novel metadevice by integrating coding metamaterials design into RME variable emissivity devices, as described in Figure 1, which enables electrically tunable IR emissivity and microwave scattering reduction. Building upon the structure of the RME variable emissivity device, “0” and “1” elements with a phase difference of ∼180° were first designed by loading Jerusalem cross (JC) metal structures. Then, by arranging the “0” and “1” elements in a chessboard-like configuration, a metadevice was formed, leading to wideband reduction of microwave scattering. Careful design considerations have resulted in a low filling ratio of the JC metal structure, minimizing the influence of the loaded JC metal structure on the IR modulation performance of the RME variable emissivity device. This ensures that the metadevice maintains the large, uniform, and consistent IR tunability in the mid-wave IR (3–5 µm) and long-wave IR (7.5–13 µm) atmospheric transmission windows [16]. The experimental results of the fabricated metadevice have further confirmed the effectiveness of this approach. Furthermore, we demonstrated the process in which the metadevice can adapt its IR emissions to blend into cold and hot backgrounds, showcasing its potential in adaptive IR camouflage. We believe that our design paves an effective path for exploring this advanced camouflage technology.

## 2 Results and discussion

### 2.1 Design of metadevice

Compared to achieving dynamic IR and microwave camouflage compatibility based on emissivity control, temperature control appears to be more readily achievable. There have been reports of integrating metamaterials for microwave camouflage into temperature control structures or devices based on microfluidic [27] or thermoelectric systems [28] to achieve this purpose. However, these approaches typically require additional heating, cooling, and pneumatic systems, inevitably leading to high energy consumption and limited flexibility in control methods. To the best of our knowledge, there are currently no reports on utilizing electrically controlled emissivity to achieve compatibility for dynamic IR and microwave camouflage. Here, we propose a realistic approach based on electrically controlled emissivity by integrating coding metamaterials into RME devices, enabling dynamic IR and microwave camouflage compatibility. Figure 1 illustrates the structure and operation principle of the proposed metadevice. The main part of the metadevice is a copper-based RME variable emissivity device, which, as an electrochemical device, features a typical sandwich structure. It utilizes a gold grid and evaporated ultra-thin platinum (Pt) film as the working electrode, and employs a copper (Cu) foil as the counter electrode, with electrolyte containing Cu^2+^ ions sandwiched between the working and counter electrodes. The electrochemical reactions involved in electrodeposition and dissolution of Cu layers in electrochromic devices can be briefly described as follows:

$$\text{C}{\text{u}}^{2+}+2{\text{e}}^{-}⇌\text{Cu}$$

Barium fluoride (BaF_2_) is employed as the IR-transparent substrate for the working electrode, onto which a JC metal structure, resembling a chessboard-like configuration, is loaded. This implies that BaF_2_ also serves as the dielectric layer in coding metamaterials, while the electrode acts as the metallic backplane layer. The integrated design scheme of metadevices incorporating coding metamaterial structures in RME devices effectively controls the overall device thickness and avoids complex structures. It is worth noting that while BaF_2_ was chosen as the IR-transparent substrate here, other IR-transparent substrates such as zinc selenide (ZnSe), silicon (Si), zinc sulfide (ZnS), and polypropylene (PP) films can also be considered. Additionally, the Cu deposition system can be adjusted to other deposition systems such as silver (Ag), bismuth (Bi), nickel (Ni), and zinc (Zn) according to requirements. However, during the design phase of metadevice structures, the following two aspects should be comprehensively considered. On the one hand, the loading of JC metal structures inevitably impacts the IR modulation performance of RME variable emissivity devices. To mitigate this impact, while ensuring outstanding microwave scattering reduction performance, efforts should be made to minimize the filling ratio of the JC metal structures on the device surface. On the other hand, the electrodeposition process unavoidably affects the device’s microwave response; hence the thickness of the electrolyte layer should be compressed to the minimum feasible extent during design to mitigate this influence (see Figure S1, Supplementary Material for detailed analysis).

**Figure 2:**
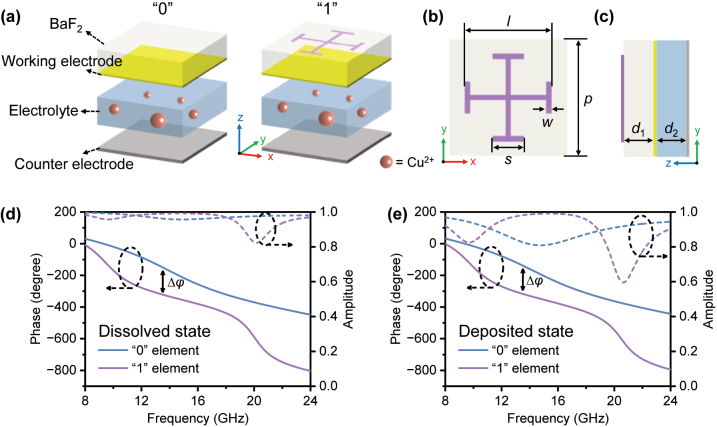
Design of “0” and “1” elements. (a) Schematic diagram of the designed “0” and “1” elements. (b) Top view and geometrical parameter of the “1” element. (c) Side view and geometrical parameter of the “1” element. (d) Simulated reflection phases and amplitudes of the “0” and “1” elements in the dissolved and (e) deposited states under normal incidence.

The key to the design of coding metamaterials lies in creating two distinct unit cells with a 180° phase difference, namely the “0” and “1” elements, respectively. Many structures, such as JC, ring, patches, and split ring, are commonly used for the design of the “0” and “1” elements. We also compare the reflection phases of different structures in Figure S2 (Supplementary Material). After considering the effective modulation bandwidth and structural filling ratio, we ultimately selected the JC structure. Figure 2(a) illustrates the schematic diagram of the designed “0” and “1” elements, with their only difference being whether or not the subwavelength JC metal structure is loaded on the RME variable emissivity device. In CST Microwave Studio, we simulated the reflection phase and magnitude of the “0” and “1” elements and optimized their structural parameters. Specifically, we set the electrolyte layer thickness *d*_2_ to a feasible minimum of 0.1 mm, and its complex permittivity was measured by the coaxial probe method (measurement results see Figure S3, Supplementary Material), and further optimized the structural parameters as depicted in Figure 2(b) and (c). The optimization of structural parameters was primarily conducted through parameter sweeping, and the results for the main geometric parameters are depicted in Figure S4 (Supplementary Materials). The geometric parameters of the structure are optimized as follows: The periodicity of the “0” and “1” elements is *p* = 4 mm; the BaF_2_ layer has a thickness of *d*_1_ = 2 mm, with a dielectric constant of 6.6 and loss tangent of 0.003; the JC metal structure layer has a thickness of *t* = 300 nm, with geometric parameters of *l* = 3.5 mm, *s* = 1.25 mm, and *w* = 0.12 mm. Additionally, it is noteworthy that the thickness of the ultra-thin Pt film is 4 nm, with a sheet resistance of ∼150 Ω/sq, and the sheet resistance of the Cu electrodeposited Pt film in the deposited state can be reduced to ∼10 Ω/sq. Based on this, we simulated the reflection phase and amplitude of the “0” and “1” elements in both the dissolved and deposited states, as illustrated in Figure 2(d) and (e). Since the Cu foil used as counter electrode has excellent conductivity and is continuous, the transmission is very small and negligible here. It can be observed that in both the dissolved and deposited states, the “0” and “1” elements exhibit an ∼180° phase difference in a wideband range of 10–20 GHz and have a high reflection amplitude. Additionally, it can be noted that the electrodeposition process does not significantly impact the microwave response of the device.

**Figure 3:**
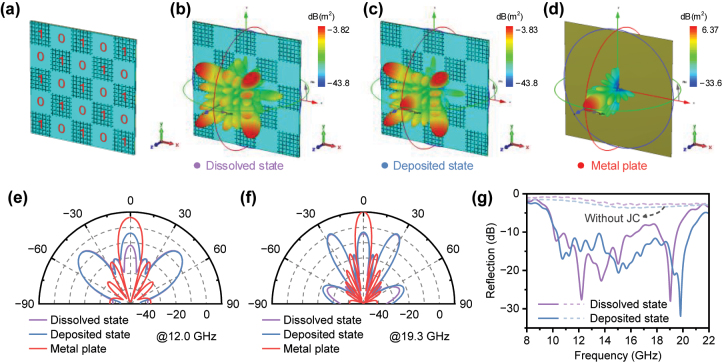
Design of metadevice. (a) Schematic diagram of the proposed metadevice with a chessboard-like configuration. (b) Simulated 3D scattering patterns under normal incidence of the metadevice in the dissolved and (c) deposited states at 12.0 GHz. (d) Simulated 3D scattering patterns under normal incidence of a metal plate with the same size as the metadevice at 12.0 GHz. (e) Simulated 2D scattering patterns under normal incidence in the 45° plane of the metadevice in the dissolved and deposited states, as well as a same-sized metal plate, at 12.0 GHz and (f) 19.3 GHz. (g) Simulated microwave reflection spectra of the metadevice in the dissolved and deposited states under normal incidence. The dashed line represents the simulated reflection spectra of the device without loading JC metal structure, i.e., RME variable emissivity device only.

After completing the design of “0” and “1” elements, we can manipulate electromagnetic waves by coding “0” and “1” elements with controlled coding sequences to achieve different functions [23]. In order to achieve the function of microwave scattering reduction, we adopted a common chessboard-like configuration here, where the coding sequence is “101,010 …” along both the *x* and *y* directions. Figure 3(a) depicts a schematic diagram of the proposed metadevices. To mitigate electromagnetic coupling between adjacent unit cells due to geometric variations, we employed a super cell consisting of 6 × 6 identical unit cells representing “0” or “1” elements. The metadevice is composed of a 5 × 5 super cell, i.e., 30 × 30 unit cells. We performed simulations to evaluate the microwave scattering reduction performance of metadevice. Figure 3(b) and (c) and Figure S5a, b (Supplementary Material) illustrate the simulated 3D scattering patterns of the metadevice in the dissolved and deposited states, respectively, under normal incidence at 12.0 and 19.3 GHz. For comparison, we also simulated the 3D scattering pattern of a metal plate with the same size (Figure 3(d) and Figure S5c, Supplementary Material). Video S1 records the simulated 3D scattering patterns of the metadevice in the deposited and dissolved states and of a metal plate with the same size at 12.0 GHz. It can be observed that the metadevice scatters incident electromagnetic waves relatively uniformly in four symmetric directions, namely 45°, 135°, 225°, and 315°, contrasting sharply with the strong backward reflection along the normal direction exhibited by the metallic plate. The simulated 2D scattering patterns in Figure 3(e) and (f) illustrate that the metadevice achieves a reflection reduction of >10 dB in the backward direction at 12.0 and 19.3 GHz compared to the metal plate. The microwave reflection spectra of the metadevice in the dissolved and deposited states are simulated as shown in Figure 3(g), and the results indicate that the metadevice maintains excellent wideband microwave scattering reduction performance in both states. Note that the bandwidth of the reduction less than −10 dB ranges approximately from 10 to 20 GHz, which is consistent with that for the phase difference. For comparison, the reflection spectra of device without loading JC metal structure, i.e., RME variable emissivity device only, were also simulated (dashed line in Figure 3(g)), and it can be observed that loading JC metal structure effectively reduces the reflection of the device. Additionally, by optimizing the arrangement of the coding sequences, a diffusion scattering effects can be achieved [29], for which further simulation analysis has been shown in Figure S6 (Supplementary Material). Furthermore, it would be interesting to explore the possibility of introducing diverse patterns for multi-state signal differentiation in the microwave wavelength range by designing different coding sequences.

### 2.2 Microwave scattering reduction performance

**Figure 4:**
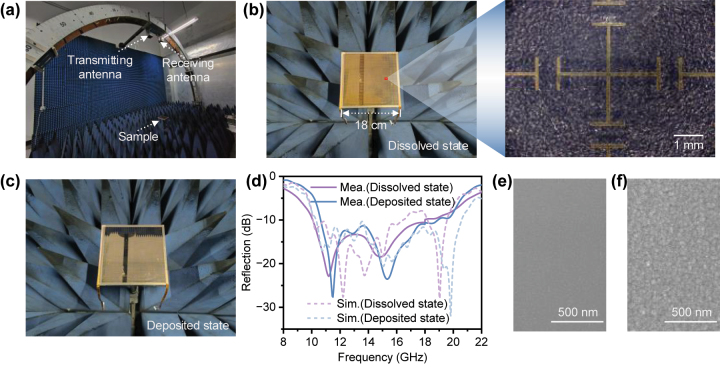
Microwave scattering reduction performance. (a) Measurement setup in a microwave anechoic chamber. (b) Photographs and micrographs of the fabricated metadevice in the dissolved state. (c) Photographs of the fabricated metadevice in the deposited state. (d) Measured microwave reflection spectra of the fabricated metadevice in the dissolved and deposited states under normal incidence. The dashed lines are simulation results. (e) SEM image of the Pt film. (f) SEM image of the Cu film formed on the Pt film in the deposited state.

To verify the microwave scattering reduction performance, we fabricated a metadevice sample with dimensions of 180 × 180 mm^2^ and conducted reflection testing in a microwave darkroom using the NRL-arc method (Figure 4(a)). Due to the high cost of large-area BaF_2_, we used glass (with a dielectric constant of 6.6 and a loss tangent of 0.003) as a substitute for BaF_2_ as the substrate for the top electrode. Since they have similar dielectric constants, this substitution has little impact on the results of the microwave reflection testing. The fabricated metadevice in the dissolved and deposited states are shown in Figure 4(b) and (c). The measured geometric parameters of the etched JC metal structure in the fabricated metadevice are shown in Figure S7 (Supplementary Material). During the measurement, the reflection from a metal plate of the same size as the sample was initially measured for normalization. The real-time microwave reflection spectra of the metadevice can then be measured by controlling the deposition time. Figure 4(d) shows the reflection spectra of the metadevice in the dissolved and deposited states, and the real-time reflection spectra of the metadevice during the electrodeposition process are shown in Figure S8 (Supplementary Material). It can be observed that throughout the electrodeposition process, the metadevice exhibits relatively stable wideband microwave scattering reduction performance in 10–20 GHz. The measurement results are relatively consistent with the simulation results, although minor discrepancies may arise due to imperfections in sample fabrication and differences between the measurement and simulation environments. Furthermore, the surface morphology of the Pt film and electrodeposited Cu film was characterized (Figure 4(e) and (f)) and it can be clearly observed that a dense and uniform Cu deposition layer was formed on the Pt film in the deposited state.

### 2.3 Dynamic IR performance

**Figure 5:**
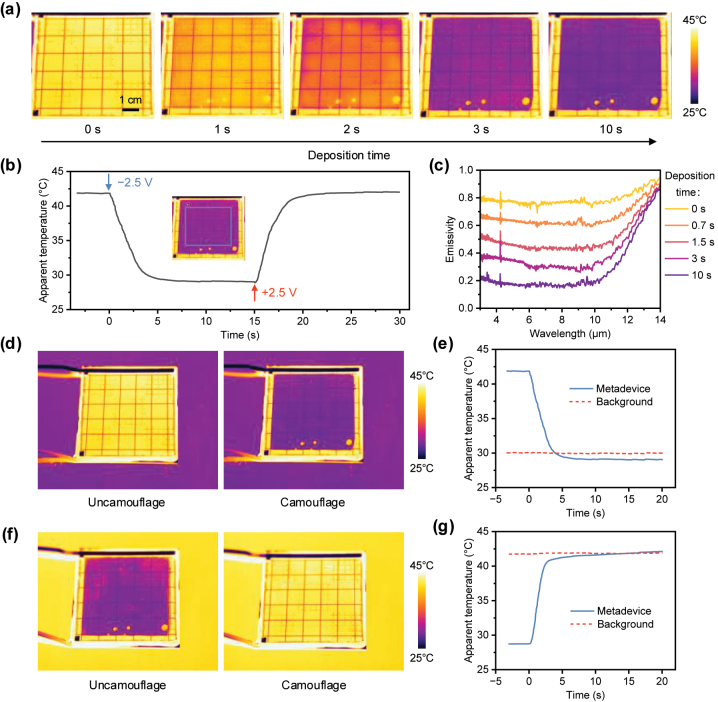
IR reversible tunability performance. (a) IR images of the fabricated metadevices during the electrodeposition process. (b) Apparent temperature curves (blue rectangular region) of the fabricated metadevices during the electrodeposition and dissolution process. (c) Real-time IR emissivity spectra of the “1” element region of the metadevice during the electrodeposition process. The general trend is increasing IR emissivity with increasing deposition time. (d–g) Adaptive IR camouflage demonstration. The device can adjust its IR emission to blend into (d) cold and (f) hot backgrounds. The graphs show the temporal evolution of the apparent temperature of the device during adaptation into (e) cold and (g) hot backgrounds with the red dashed line representing the apparent temperature of the background.

To verify the dynamic IR performance, we fabricated a metadevice sample with dimensions of 70 × 70 mm^2^ and recorded IR video of the fabricated metadevice during the electrodeposition and dissolution process using an IR thermal imager, as shown in Video S2 (Supplementary Material), and captured a series of IR images from the video, as shown in Figure 5(a) and Figure S9 (Supplementary Material). Figure 5(b) illustrates the apparent temperature curves of the metadevice during the electrodeposition and dissolution process. The real temperature of the metadevice was controlled at 50 °C using a hot plate during the experiment. Therefore, the change in apparent temperature implies a change in the IR emissivity of the metadevice. When the deposition voltage was applied, Cu film was gradually electrodeposited on the working electrode, and as the deposition time increased, the Cu film became thicker, leading to a corresponding decrease in the apparent temperature of the sample. When the dissolution voltage was applied, the deposited Cu film gradually dissolved until the initial state was restored, demonstrating reversibility. Due to the long-distance conductivity provided by the gold grid, the device exhibited relatively uniform IR modulation capability during the electrodeposition and dissolution process. In addition, we measured the real-time IR reflection spectra of the “0” element and “1” element regions of the sample (Figure S10, Supplementary Material) and calculated the real-time IR emissivity spectra (Figure 5(c) and Figure S11, Supplementary Material). Although the surface of the “1” element region of the device is covered with a JC metal structure array, its relatively small filling rate results in a limited impact on the IR emissivity modulation performance compared to the “0” element region. By integrating the spectral data, we calculated the maximum emissivity modulation of the metadevice in mid-wave IR and long-wave IR atmospheric transmission windows to be 0.58 and 0.50, respectively, demonstrating a relatively consistent level of emissivity modulation. Notably, various electrochromic materials, including those based on RME, have been reported to achieve reversible tuning in the near-IR band, which is an important guide for further exploration to broaden the tuning band [30], [31], [32], [33].

To showcase the prospects of the metadevice, we conducted a demonstration of its adaptive IR camouflage potential in a laboratory setting. Samples with different emissivity were placed on a hot plate at 50 °C to create distinct IR backgrounds. The metadevice was then placed on each background, and the process of the metadevice actively blending with the background was recorded using an IR thermal imager, as shown in Video S3 and Video S4 (Supplementary Material). Figure 5(d) shows IR images of the metadevice as it adapts its IR emission to cold backgrounds. Here, ITO glass with a sheet resistance of 15 Ω/sq was utilized as the cold background. In the dissolved state (initial state), the device exhibited a high IR emission state, resulting in an apparent temperature significantly higher than the background, making it prone to exposure. By applying the deposition voltage, the metadevice can switch to a low IR emission state, effectively blending into the cold background environment and achieving camouflage. Similarly, we utilized glass as the hot background, and presented IR images of the metadevice as it adapts its IR emission to hot backgrounds in Figure 5(f). The temporal evolution of the apparent temperatures of the metadevice and background, as shown in Figure 5(e), (g), not only quantifies the adaptation of the device’s IR emission to the background, but also reveals its high transition speed (∼4 s). In fact, by precisely controlling the deposition time, placing the metadevice in an intermediate emissivity state enables integration with more IR backgrounds. Furthermore, by incorporating perception and control systems into the metadevice, human intervention can be avoided, enhancing the device’s adaptive capabilities [34]. Considering the adoption of pixelated architecture design [35], [36], segmented addressing techniques [37], and intelligent control algorithms [26] to achieve pixelized addressing control in devices, is also crucial for the development of devices in fields such as IR digital camouflage and information display.

## 3 Conclusions

In summary, by integrating RME and coding metamaterials, a novel multispectral metal-based electro-optical metadevice capable of simultaneous electrically tunable IR emissivity and microwave scattering reduction is proposed. The combination of metamaterials with extraordinary electromagnetic wave manipulation capabilities and emerging electrochromic technologies is bound to spark more innovation. Our research is expected to stimulate academic interest in the integration of metamaterials with established visible or IR electrochromic materials and devices, thereby driving the advancement of advanced functional material systems with multispectral adaptive camouflage capabilities. In the future, our focus will be on enhancing the stability of the devices and achieving large-scale flexibility, with the expectation of broader applications for this technology.

## 4 Experimental section

### 4.1 Simulations

Full-wave simulations of the metadevice were carried out using the time-domain solver in CST Microwave Studio 2022. The incident plane waves were normally incident upon the devices from +*Z* direction. The boundaries along *x*, *y* and *z* directions were set as open add space. Far-field monitor is set at 12.0 GHz and 19.3 GHz to get the scattering pattern.

### 4.2 JC metal structure preparation

A gold film with a thickness of 300 nm was initially evaporated on BaF_2_ substrates using an electron-beam evaporation system (Kurt J. Lesker PVD 75) with a deposition rate of 0.08 nm s^−1^ at room temperature, and then the gold film was etched using a laser etcher (SC-K750) to form the JC metal structure.

### 4.3 Working electrode preparation

The BaF_2_ substrates with a thickness of 2 mm were used as the working electrode substrates. The gold grid with 200 nm line thickness, 300 µm line width, and 12 mm line spacing was directly evaporated on the BaF_2_ substrates with the aid of a stainless steel mask plate using an electron-beam evaporation system (Kurt J. Lesker PVD 75) with a deposition rate of 0.08 nm s^−1^ at room temperature, and then the Pt film with a thickness of 4 nm was further evaporated with a deposition rate of 0.02 nm s^−1^ at room temperature. The nominal thicknesses of the gold grid and Pt film were determined by extrapolation of the deposition rate, in turn calibrated using a quartz crystal oscillator.

### 4.4 Gel electrolyte preparation

The gel electrolyte was prepared by mixing 0.08 M CuCl_2_ (Aladdin), 0.25 M LiClO_4_ (Aladdin), 0.6 mM KI (Aladdin), and 10 wt% PVB (Sinopharm Chemical Reagent) in DMSO (Aladdin) solution. All chemicals were mixed and stirred on a hot plate set to 60 °C and 900 rpm for at least 12 h.

### 4.5 Device assembly

The metadevice was constructed by using BaF_2_ substrates with evaporated gold grid and Pt film as the working electrode. Cu foil with a thickness of 0.05 mm was used as the counter electrode. The perimeters of the working and counter electrodes were framed with Cu tape to provide uniform electrical contacts. Polyamide tapes were used to seal the Cu tape and prevent contact with the electrolyte. A double-sided tape with a thickness of 0.1 mm was used to seal the edges of the electrodes, which worked as the frame of the devices. Silicone adhesive sealant was applied to the edges of the devices. The electrolyte was subsequently injected into the device.

### 4.6 Characterization

The sheet resistance of the evaporated Pt films was measured using a four-probe resistivity measurement system (RTS-9). The complex permittivity of gel electrolyte was measured using a dielectric performance probe (Keysight N1501A) connected to a network analyzer (Agilent E8363C). The microwave reflection measurements were conducted using broadband standard gain horn antennas connected to a network analyzer (Agilent E8363C). The samples were photographed using a digital camera (Canon EOS M50 Mark II). The SEM images were obtained with field-emission scanning electron microscope (MIRA3 AMU). The IR reflection spectra (3–14 µm) were measured using a Fourier transform IR (FTIR) spectrometer (Bruker Vertex 70) equipped with a mid-IR integrating sphere (A562). The IR images were recorded using an IR thermal imager with a working range of 7.5–14 µm (FLIR T1050sc), with predefined emittances set to 0.95. The apparent temperature curves the devices in IR images were extracted by the box measurement tool in the FLIR software packages (FLIR Tools V 5.7). A PARSTAT 4000 Advanced Electrochemical System (Princeton Applied Research, USA) was used for electrochemical measurements and device performance demonstration.

## Supplementary Material

Supplementary Material Details

Supplementary Material Details

Supplementary Material Details

Supplementary Material Details

Supplementary Material Details

Supplementary Material Details

Supplementary Material Details

Supplementary Material Details

Supplementary Material Details

## References

[1] Wu Y., Tan S., Zhao Y., Liang L., Zhou M., Ji G. Broadband multispectral compatible absorbers for radar, infrared and visible stealth application. *Prog. Mater. Sci*..

[2] Zhu H. (2021). Multispectral camouflage for infrared, visible, lasers and microwave with radiative cooling. *Nat. Commun.*.

[3] Lee N., Lim J.-S., Chang I., Bae H. M., Nam J., Cho H. H. (2022). Flexible assembled metamaterials for infrared and microwave camouflage. *Adv. Opt. Mater.*.

[4] Wen C. (2022). High-density anisotropy magnetism enhanced microwave absorption performance in Ti3C2Tx MXene@Ni microspheres. *ACS Nano*.

[5] Wu Y. (2022). Ultrabroad microwave absorption ability and infrared stealth property of nano-micro CuS@rGO lightweight aerogels. *Nano-Micro Lett.*.

[6] Tian H., Liu H.-T., Cheng H.-F. (2014). A thin radar-infrared stealth-compatible structure: design, fabrication, and characterization. *Chin. Phys. B*.

[7] An Z., Li Y., Luo X., Huang Y., Zhang R., Fang D. (2022). Multilaminate metastructure for high-temperature radar-infrared bi-stealth: topological optimization and near-room-temperature synthesis. *Matter*.

[8] Feng X. (2022). Large-area low-cost multiscale-hierarchical metasurfaces for multispectral compatible camouflage of dual-band lasers, infrared and microwave. *Adv. Funct. Mater.*.

[9] Kim T., Bae J.-Y., Lee N., Cho H. H. (2019). Hierarchical metamaterials for multispectral camouflage of infrared and microwaves. *Adv. Funct. Mater.*.

[10] Hao K. Design of one-dimensional composite photonic crystal with high infrared reflectivity and low microwave reflectivity. *Optik*.

[11] Ergoktas M. S. (2020). Graphene-Enabled adaptive infrared textiles. *Nano Lett.*.

[12] Hong S., Shin S., Chen R. (2020). An adaptive and wearable thermal camouflage device. *Adv. Funct. Mater.*.

[13] Yang J., Zhang X., Zhang X., Wang L., Feng W., Li Q. (2021). Beyond the visible: bioinspired infrared adaptive materials. *Adv. Mater.*.

[14] Hu R. (2021). Thermal camouflaging metamaterials. *Mater. Today*.

[15] Jia Y. (2023). Transparent dynamic infrared emissivity regulators. *Nat. Commun.*.

[16] Li M., Liu D., Cheng H., Peng L., Zu M. (2020). Manipulating metals for adaptive thermal camouflage. *Sci. Adv.*.

[17] Tao X., Liu D., Liu T., Meng Z., Yu J., Cheng H. (2022). A bistable variable infrared emissivity device based on reversible silver electrodeposition. *Adv. Funct. Mater.*.

[18] Rao Y. (2021). Ultra-wideband transparent conductive electrode for electrochromic synergistic solar and radiative heat management. *ACS Energy Lett.*.

[19] Schurig D. (2006). Metamaterial electromagnetic cloak at microwave frequencies. *Science*.

[20] Landy N. I., Sajuyigbe S., Mock J. J., Smith D. R., Padilla W. J. (2008). Perfect metamaterial absorber. *Phys. Rev. Lett.*.

[21] Zhu R. (2021). Phase-to-pattern inverse design paradigm for fast realization of functional metasurfaces via transfer learning. *Nat. Commun.*.

[22] Qiu T. (2024). Vision-driven metasurfaces for perception enhancement. *Nat. Commun.*.

[23] Cui T. J., Qi M. Q., Wan X., Zhao J., Cheng Q. (2014). Coding metamaterials, digital metamaterials and programmable metamaterials. *Light: Sci. Appl.*.

[24] Ma Q., Cui T. J. (2020). Information Metamaterials: bridging the physical world and digital world. *PhotoniX*.

[25] Modi A. Y., Balanis C. A., Birtcher C. R., Shaman H. N. (2017). Novel design of ultrabroadband radar cross section reduction surfaces using artificial magnetic conductors. *IEEE Trans. Antennas Propag.*.

[26] Qian C. (2020). Deep-learning-enabled self-adaptive microwave cloak without human intervention. *Nat. Photonics*.

[27] Wen J., Ren Q., Peng R., Zhao Q. (2022). Multi-functional tunable ultra-broadband water-based metasurface absorber with high reconfigurability. *J. Phys. D: Appl. Phys.*.

[28] Yuan L. M. (2022). A dynamic thermal camouflage metadevice with microwave scattering reduction. *Adv. Sci.*.

[29] Meng Z. (2020). Optically transparent coding metasurface with simultaneously low infrared emissivity and microwave scattering reduction. *Opt. Express*.

[30] Eh A. L.-S. (2020). A quasi-solid-state tristate reversible electrochemical mirror device with enhanced stability. *Adv. Sci.*.

[31] Ionescu A. (2016). Near-IR electrochromism in electrodeposited thin films of cyclometalated complexes. *ACS Appl. Mater. Interfaces*.

[32] Ko J. H., Seo D. H., Jeong H.-H., Kim S., Song Y. M. (2024). Sub-1-Volt electrically programmable optical modulator based on active tamm plasmon. *Adv. Mater.*.

[33] Karst J. (2021). Electrically switchable metallic polymer nanoantennas. *Science*.

[34] Zhu R. Chameleon-like intelligent camouflage metasurface. *Mater. Des.*.

[35] Ergoktas M. S. (2021). Multispectral graphene-based electro-optical surfaces with reversible tunability from visible to microwave wavelengths. *Nat. Photonics*.

[36] Sun Y. (2021). Large-scale multifunctional carbon nanotube thin film as effective mid-infrared radiation modulator with long-term stability. *Adv. Opt. Mater.*.

[37] SSalihoglu O. (2018). Graphene-based adaptive thermal camouflage. *Nano Lett.*.

