# Supplementary material for: Multispectral metal-based electro-optical metadevices with infrared reversible tunability and microwave scattering reduction
Source: Nanophotonics. 2024 May 22;13(17):3165–74. doi: 10.1515/nanoph-2024-0202 (PMC11501323; doi:10.1515/nanoph-2024-0202)
Supplement: Supplementary file 1 — Supplementary Material Details [file j_nanoph-2024-0202_suppl_001.docx]

Supplementary Material:

Multispectral metal-based electro-optical metadevices with infrared reversible tunability and microwave scattering reduction

Zhen Meng,^1^ Dongqing Liu, ^1,*^ Yongqiang Pang,^2^ Jiafu Wang,^3^ Tianwen Liu,^1^ Yan Jia,^1^ Haifeng Cheng^1^

*^1^Science and Technology on Advanced Ceramic Fibers and Composites Laboratory, College of Aerospace Science and Engineering, National University of Defense Technology, Changsha 410073, China*

*^2^ Electronic Materials Research Laboratory, Key Laboratory of the Ministry of Education, Xi’an Jiaotong University, Xi’an 710049, China*

*^3^Shaanxi Key Laboratory of Artificially-Structured Functional Materials and Devices, Air Force Engineering University, Xi’an 710051, China*

**Corresponding author: Dongqing Liu (Email: liudongqing07@nudt.edu.cn)*

**1. Calculation of the Infrared Emissivity**

The band emissivity of the metadevice was calculated by integrating the total spectral emissivity over the blackbody radiation spectra range:

$\varepsilon(\lambda_{1},\lambda_{2})=\frac{\int_{\lambda_{1}}^{\lambda_{2}} I_{BB}(T,\lambda)\varepsilon(T,\lambda)d\lambda}{\int_{\lambda_{1}}^{\lambda_{2}} I_{BB}(T,\lambda)d\lambda}$ (S1)

where *λ* is the wavelength, $(\lambda_{1},\lambda_{2})$ is the bandwidth of interest, $I_{BB}(T,\lambda)$ is the spectral intensity emitted by a blackbody at temperature *T* (assumed to be 298 K) and $\varepsilon(T,\lambda)$ is the spectral emissivity of the devices at temperature *T* and wavelength *λ*. According to Kirchhoff’s rule for objects at thermal equilibrium, $\varepsilon(T,\lambda)$ is equivalent to $\alpha(T,\lambda)$, where $\alpha(T,\lambda)$ is the spectral absorptivity at temperature *T* and wavelength *λ*. And for infrared opaque objects, it can be expressed as $100\% -R(T,\lambda)$ , where $R(T,\lambda)$ is the spectral reflectivity at temperature *T* and wavelength *λ*.

**2. Supplementary Figures**

**Fig. S1:** (a) Simulated reflection phases and amplitudes of the “0” and “1” elements with electrolyte layer thickness of 1 mm in the dissolved and (b) deposited states under normal incidence. (c) Simulated microwave reflection spectra of the device with electrolyte layer thickness of 1 mm in the dissolved and deposited states under normal incidence.

**Note:** To validate the significance of controlling the electrolyte layer thickness in mitigating the impact of electrodeposition process on device microwave response, we conducted simulations with an electrolyte layer thickness of 1 mm to compare it with a thickness of 0.1 mm (i.e., our proposed metadevice). As shown in Figure S1a,b, when the electrolyte layer thickness is 1 mm, significant differences in reflection phase and amplitude are observed for “0”and “1” elements in dissolved and deposited states. Specifically, in dissolved state, a reflection phase difference of ~180° is observed only at 8–11 GHz, accompanied by a significant reduction in reflection amplitude due to enhanced absorption by “0”and “1” elements. In contrast, in deposited state, the changes in reflection phase and magnitude are minimal compared to when the electrolyte layer thickness is 0.1 mm (see Figure 2e). This inevitably leads to inconsistent microwave scattering performance between the dissolved and deposited states. Additionally, we also simulated the reflection spectra of the devices in a chessboard-like configuration with a 1 mm electrolyte layer thickness, as shown in Figure S1c, and the results indicate significant differences in the microwave reflection spectra of the device before and after electrodeposition. These analyses further confirm the effectiveness of controlling electrolyte layer thickness in mitigating the impact of the electrodeposition process on device’s microwave response.

**Fig. S2:** (a) Simulated reflection phases of unit cells with different sizes of Jerusalem cross (JC), (b) ring, (c) patch, and different rotation angles of (d) Z-shaped and (e) split ring (SR) in the deposited state under normal incidence. (f) Phase difference between unit cells. The grey area represents a phase difference range from 143° to 217°. It can be seen that the phase difference between the two unit cells using JC structure has the widest bandwidth in the grey area.

**Fig. S3:** Complex permittivity of the gel electrolyte measured by coaxial probe method.

**Fig. S4:** The parameter sweeping results for the main geometric parameters.

**Note:** Since the reflection amplitude of “0” and “1” elements has remained consistently high, with minimal impact on the scattering performance, the focus during parameter scanning has primarily been on the reflection phase results. To facilitate the observation of phase changes during the parameter scanning of main geometric parameters, we have kept the “0” element structure unchanged and displayed the parameter scanning results for the main geometric parameters of the “1” elements. As shown in Figure S4, the reflection phase of the “1” element is most sensitive to the *l* value of the JC structure and least sensitive to the *w* value of the JC structure. The final optimized geometric parameters ensure that the “0” and “1” elements achieve a ~180° phase difference across as wide a frequency band as possible.

**Fig. S5:** (a) Simulated 3D scattering patterns under normal incidence of the metadevice in the dissolved and (b) deposited states at 19.3 GHz. (c) Simulated 3D scattering patterns under normal incidence of a metal plate with the same size as the metadevice at 19.3 GHz.

**Fig. S6:** (a) Schematic diagram of the metadevice with an optimized coding sequence configuration. (b) Simulated 3D scattering patterns under normal incidence of the metadevice in the dissolved state at 10 GHz, (c) 14 GHz, and (d) 18 GHz. (e) Simulated 3D scattering patterns under normal incidence of the metadevice in the deposited state at 10 GHz, (f) 14 GHz, and (g) 18 GHz. (h) Simulated 3D scattering patterns under normal incidence of a metal plate with the same size as the metadevice at 10 GHz, (i) 14 GHz, and (j) 18 GHz. (k) Simulated 2D scattering patterns under normal incidence in the 45° plane of the metadevice in the dissolved and deposited states, as well as a same-sized metal plate, at 10 GHz, (l) 14 GHz, and (m) 18 GHz.

**Fig. S7:** Measured geometric parameters of the etched JC metal structure.

**Fig. S8:** Measured real-time microwave reflection spectra of the metadevice in 8–18 GHz during the electrodeposition process under normal incidence.

**Fig. S9:** Infrared images of the fabricated metadevices during the dissolution process.

**Fig. S10:** (a) Real-time infrared reflection spectra of the “0” element and (b) “1” element regions of the metadevice during the electrodeposition process.

**Fig. S11:** Real-time infrared emissivity spectra of the “0” element region of the metadevice during the electrodeposition process.

**3. Supplementary Videos**

**Video S1**: Simulated 3D scattering patterns of the metadevice in the deposited and dissolved states and of a metal plate with the same size at 12.0 GHz.

**Video S2**: Infrared thermal movie of the metadevice during electrodeposition and dissolution.

**Video S3**: Video showing adaptation of the infrared appearance to a cold background.

**Video S4**: Video showing adaptation of the infrared appearance to a hot background.
